# Who benefits most?

**DOI:** 10.1192/j.eurpsy.2021.85

**Published:** 2021-08-13

**Authors:** L. Van Diermen

**Affiliations:** Old Age Psychiatry, PC Bethanië, Zoersel, Belgium

**Keywords:** Electroconvulsive therapy, Outcome predictors

## Abstract

We know from past meta-analyses that several clinical variables are associated with electroconvulsive therapy (ECT) outcome in major depression. In this lecture we give an update of clinical variables associated with ECT outcome and dig deeper into the fact that these variables also seem to be somehow associated with each other. We attempt to disentangle the interdependence between the clinical variables and try to distil the most important predictors of treatment success to help improve patient-treatment matching. Therefore we created a conceptual framework of interdependence between predictors capturing age, episode duration, and treatment resistance, all variables associated with ECT outcome, and the clinical symptoms of what we have called ‘core depression’, i.e., depression with psychomotor agitation, retardation, or psychotic features, or a combination of the three. We validated this model in a sample 73 patients using path analyses, with the size and direction of all direct and indirect paths being estimated using structural equation modelling. Results of these analyses were recently published and will also be disscussed at this symposium. The conceptual model could eb largely validated, the most important finding being that age was only indirectly associated with ECT outcome, meaning that age seems to be associated with ECT outcome only because more psychomotor and psychotic symptoms occur in elderly patients with a depressive disorder.

**Disclosure:**

No significant relationships.

